# Root dentin surface activation to improve bioceramic bonding: A scanning electron microscopic study

**DOI:** 10.34172/joddd.2020.025

**Published:** 2020-06-17

**Authors:** Shazeena Qaiser, Mithra N. Hegde, Darshana Devadiga, Mahalaxmi Yelapure

**Affiliations:** ^1^Department of Conservative Dentistry & Endodontics, A B Shetty Memorial Institute of Dental Sciences

**Keywords:** Bioceramic, Surfactants, bonding, Wettability, Marginal adaptation

## Abstract

**Background.** Bioceramics need to interact chemically with dentin to exhibit adequate bioactivity. Proper bonding of bioceramics with dentin is of considerable importance. This study aimed to evaluate the wettability and marginal adaptation of bioceramics after the use of surface-active agents on dentin: %0.5 cetrimide and %1 alkylbenzene sulfonate.

**Methods.** Of ninety maxillary central incisors, 45 teeth were sectioned longitudinally with 45 root halves randomly assigned to three groups (n=15): group I: no pre-treatment; group II: %0.5 cetrimide; group III: %1 alkylbenzene sulfonate. Then, the samples were subdivided into three subgroups (n=5): subgroup I: MTA; subgroup II: Biodentine, and subgroup III: BioRoot. A controlled-volume droplet of bioceramic material was placed on each root half, which was positioned in a dynamic contact angle analyzer to record the static contact angle for wettability. The remaining 45 samples were decoronated; the root canals were prepared and randomly categorized, as mentioned above. The root canal surfaces were treated, filled with the bioceramic material, transversely sectioned, and then each middle section was analyzed microscopically for marginal adaptation. Statistical tests used included post hoc Tukey tests and one-way ANOVA. The level of statistical significance set at %95 (P<0.05).

**Results.** The contact angle values and interfacial gap width values after surface pre-treatment were significantly lower when compared to no pre-treatment group (P<0.05). The values were significant for %0.5 cetrimide in the case of Biodentine and %1 alkylbenzene sulfonate in the case of BioRoot (P<0.05).

**Conclusion.** The two surfactants yielded promising results for enhancing the wettability and marginal adaptation of materials to the root dentin, which is required for obtaining an adequate seal, penetration, and bond strength of bioceramics.

## Introduction

There is an increasing interest in the application of bioceramics in medical and dental fields. The development of bioceramic-based technology is one of the fascinating innovations in the field of material science. Bioceramics have perfectly combined the sealing ability and biocompatibility and exhibited favorable characteristics and promising results.^1^ However, in our last study, where dentinal penetration of BioRoot RCS was evaluated and compared with the gold standard AH plus, SEM images demonstrated many interfacial gaps for BioRoot RCS.

The interfacial adaptation of bioceramics is imperative, whether it be used as a pulp capping agent, root-end filling material or as a root canal sealer since they need to interact chemically with dentin to exhibit adequate bioactivity.^2^ Therefore, the proper wettability of the radicular dentin with bioceramics and contact angle between the material and the hard tissue should be considered. This study was designed to evaluate the wettability for bioceramics after dentin surface pre-treatment with surfactants, which might influence its surface adaptation.

A practical indicator for wettability is the contact angle which is formed between a material (liquid) and a dentin surface (solid).^3-6^ The contact angle is inversely related to wettability and surface free energy. A surface with a low contact angle exhibits greater wettability compared to a substance with a higher contact angle. Contact angle also influences the spreading and adsorption of liquids;^7,8^ the smaller the contact angle, the better the adhesion to dentin surface.^9,10,11^ Another property which reflects the sealing capacity is the marginal adaptation of bioceramics with the dentin, which was evaluated using scanning electron microscopy (SEM).^12^

The surfactants exist in three categories: anionic, cationic, or non-ionic. Cetrimide (0.5%) is chemically cetyltrimethylammonium bromide (CTAB), a quaternary ammonium compound, used as a cationic surfactant and a disinfecting agent as well. It has been reported to reduce the surface tension of the irrigant, increase antibacterial effectiveness, enhance the penetration of the irrigant to the dentin surface, and increases the wettability of the dentin surface.^13-17^ Fehr and Nygaard-Ostby suggested adding 0.84 gr of a quaternary ammonium bromide (Cetavlon or Cetrimide) to transform EDTA to EDTAC, which reduces the surface tension and increases the penetration capacity of the solution.^18^ However, it has rarely been reported to be used as a surfactant alone on the dentinal surface. Alkylbenzene sulfonate belongs to a group of anionic surfactants consisting of a hydrophilic sulfonate head-group and a hydrophobic alkylbenzene tail group. Along with sodium laureth sulfate, they are one of the oldest and most widely used synthetic detergents.^19^ To date, no study has assessed the effectiveness of alkylbenzene sulfonate as a surfactant on the root dentin. Also, to date, no study has evaluated the contact angle and marginal adaptation of bioceramics after dentin pre-treatment with the surfactants mentioned above.

Hence, this study was designed to evaluate the wettability and marginal adaptation of bioceramics after dentin surface pre-treatment with cetrimide and alkylbenzene sulfonate.

## Methods

### Materials used

The two surfactants used were 0.5% cetrimide (Cetrilak Menarini India Pvt Ltd) and 1% alkylbenzene sulfonate (Labsa Chemical, Vapi Surfactants, India) for one minute.

**Dilution:** To prepare a 0.5% cetrimide solution out of 5% cetrimide (commercially available), it was mixed with 1000 mL of water; to prepare 1% alkylbenzene sulfonate out of the 96% solution (commercially available), it was mixed with 9600 mL of water.

*Three bioceramic materials [MTA Angelus*  (Angelus, Londrina, Brazil); Biodentine (Septodont, Saint Maur Des Fosses, France); BioRoot RCS (Septodont, Saint Maur Des Fosses, France)] were used in this study.

### Sample selection

Ninety maxillary central incisors were collected and disinfected according to the recommendations and guidelines laid by the Occupational Safety and Health Administration (OSHA). The samples were evaluated and selected based on the inclusion criteria and cleaned of soft tissue and calculi using an ultrasonic device.

### Preparation of the specimens

**Figure 1:**
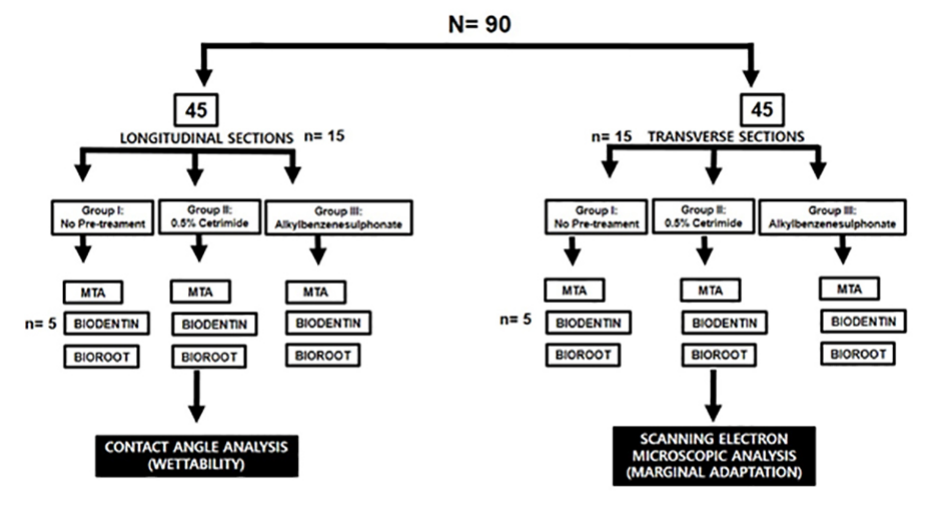
Brief illustration of the study design.

All the samples were divided into two groups (n=45 each). The first 45 samples were made into longitudinal sections for contact angle measurement, and the remaining 45 were prepared and transversely cross-sectioned to evaluate marginal adaptation using a scanning electron microscope. The samples were categorized, as illustrated in Figure 1.

### Sample preparation for contact angle analysis

**Figure 2:**
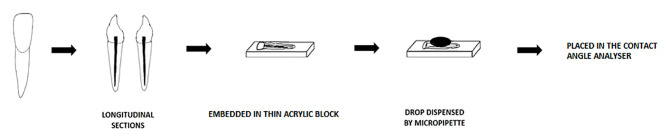
Sample preparation for contact angle analysis.

Forty-five samples were sectioned longitudinally in a buccolingual direction with a diamond disk (Confident Dental Equipments Ltd., India) under water irrigation. The two halves were appropriately examined, and only one of the two was used. Each longitudinal section was mounted on thin acrylic blocks. The dentin surface was ground with wet 100-grit sandpaper and then polished with 400- and 600-grit sandpaper. The samples were subjected to pre-treatment with surfactants for one minute, rinsed, and dried; then, each specimen was positioned on the glass slide in the analyzer. Simultaneously, the bioceramic materials were mixed, and a controlled volume was dispensed with a micropipette onto the treated root dentin surface and allowed to settle for 60 seconds (Eppendorf Reference, Adjustable-volume, Eppendorf AG, Hamburg, Germany) (Figure 2).

### Contact angle ,easurement

The contact angle was measured by a dynamic contact angle analyzer, FTA 200 (First Ten Angstroms, Portsmouth, VA, USA), using FTA software.

### Sample preparation for scanning electron microscopic examination

**Figure 3:**
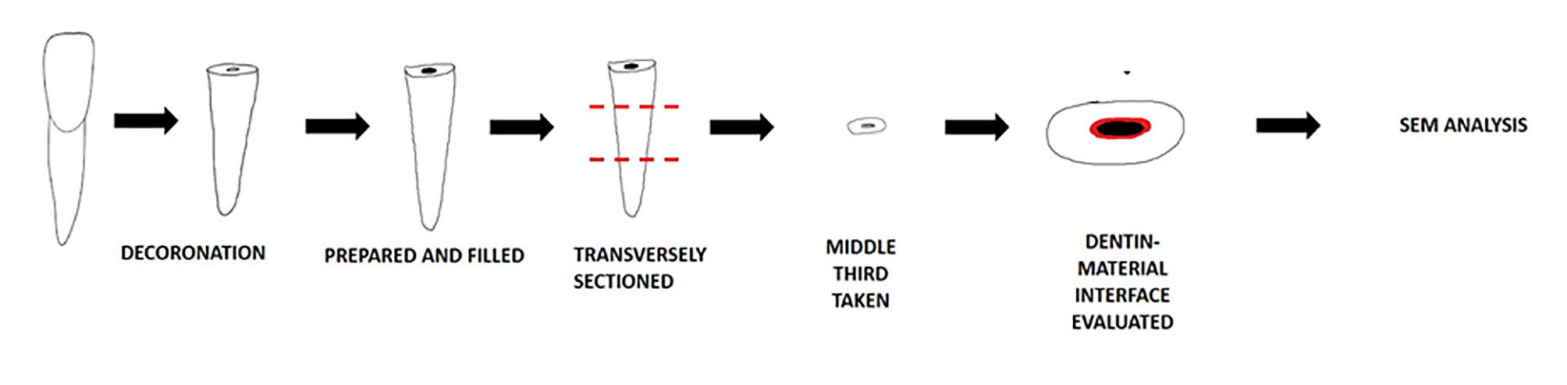
Sample preparation for Scanning Electron Microscopic analysis

The remaining 45 samples were decoronated using a diamond disc under copious water irrigation to achieve root sections. The working length was determined using a #10 K-file inserted into the root canal until the tip was visible at the apex; one millimeter was subtracted from this length to determine the working length. The root canals were prepared using ProTaper rotary files up to F3 (30/0.09). The root canals were rinsed with a 5.25% sodium hypochlorite (NaOCl) solution between the filing. A final rinse was carried out with 5 mL of distilled water, and the root canals were then dried with paper points. The samples were subjected to surface pre-treatment as mentioned; each specimen was then rinsed and dried. The three bioceramic materials were then mixed according to the manufacturers’ instructions and placed inside the root canal with a 23 gauge needle until the canal was filled. The teeth were stored in 100% humidity at 37°C for seven days for complete setting. Then, the roots were transversely split using a diamond disc into three sections: coronal, middle, and apical (Figure 3).

### Scanning electron ,icroscopic analysis 

The cut sections were dehydrated for observation by SEM. Following mounting on aluminum stubs, they were coated with a thin layer of gold in a coater system. Under the SEM, two to three representative areas from the middle third of each sample were focused, and interfacial gaps were measured using Image J software.

### Statistical analysis

All the measurements, i.e., contact angle values and interfacial gap widths, were tabulated, and the data were analyzed. Two-way ANOVA was used to compare the contact angles and interfacial gaps between the sub-groups. Post hoc Tukey tests were used for pairwise comparisons between subgroups and the main groups. Pearson’s correlation test was used to check for the correlation between wettability and marginal adaptation. The level of statistical significance was set at 95% (P<0.05).

## Results

Tables 1 and 2 present the mean and standard deviation values of contact angles and interfacial gap widths after pre-treatment of the root dentin with the surfactants 0.5% cetrimide and 1% alkylbenzene sulfonate in the three bioceramic materials, respectively. The surfactants used significantly affected the wettability and marginal adaptation of MTA, Biodentine, and BioRoot RCS (P<0.001). For MTA, there was no significant difference between the contact angles and interfacial gap width produced on cetrimide-treated root dentin and alkylbenzene sulfonate-treated root dentin. For Biodentine, contact angles and interfacial gap width values for cetrimide-treated root dentin were significantly lower than those for alkylbenzene sulfonate-treated root dentin (P<0.05). For BioRoot RCS, contact angles and interfacial gap width values for alkylbenzene sulfonate-treated root dentin were significantly lower than those for cetrimide-treated root dentin (P<0.05) (Table 3, 4). However there was no correlation seen between the wettability and marginal adaptation of the bioceramic. (Table 5)

**Table 1:** Comparison of the contact angles between the subgroups in each study group

**Group**	**Subgroup**	**N**	**Mean**	**SD**	**ANOVA**	
					**F**	**P-value**
**1**	**MTA**	5	68.20	0.84	77.84	<0.001*
	**Biodentine**	5	64.40	0.89		
	**BioRoot**	5	61.00	1.00		
**2**	**MTA**	5	57.80	1.30	74.10	<0.001*
	**Biodentine**	5	49.20	0.84		
	**BioRoot**	5	51.20	1.30		
**3**	**MTA**	5	55.40	0.55	255.18	<0.001*
	**Biodentine**	5	54.80	0.84		
	**BioRoot**	5	45.80	0.84		

*P<0.05, statistically significant; P>0.05, not significant, NS

**Table 2:** Comparison of interfacial gap width between the subgroups in each study group

**Group**	**Subgroup**	**N**	**Mean**	**SD**	**ANOVA**	
					**F**	**P-value**
**1**	**MTA**	5	24.80	1.92	77.47	<0.001*
	**Biodentine**	5	14.40	1.14		
	**BioRoot**	5	18.60	0.55		
**2**	**MTA**	5	7.40	1.14	5.24	0.02*
	**Biodentine**	5	5.40	0.55		
	**BioRoot**	5	6.60	1.14		
**3**	**MTA**	5	9.80	0.84	36.78	<0.001*
	**Biodentine**	5	7.60	0.89		
	**BioRoot**	5	5.60	0.55		

*P<0.05, statistically significant; P>0.05, not significant, NS

**Table 3:** Comparison of the contact angle between the study groups in each subgroup

**Subgroup**	**Groups**	**N**	**Mean**	**SD**	**ANOVA**	
					**F**	**P-value**
**MTA**	**1**	5	68.20	0.84	257.19	<0.001*
	**2**	5	57.80	1.30		
	**3**	5	55.40	0.55		
**Biodentine**	**1**	5	64.40	0.89	402.91	<0.001*
	**2**	5	49.20	0.84		
	**3**	5	54.80	0.84		
**BioRoot**	**1**	5	61.00	1.00	261.94	<0.001*
	**2**	5	51.20	1.30		
	**3**	5	45.80	0.84		

*P<0.05, statistically significant; P>0.05, not significant, NS

**Table 4:** Comparison of interfacial gap width between the study groups in each subgroup

**Subgroup**	**Groups**	**N**	**Mean**	**SD**	**ANOVA**	
					**F**	**P-value**
**MTA**	1	5	24.80	1.92	234.00	<0.001*
	2	5	7.40	1.14		
	3	5	9.80	0.84		
**Biodentine**	1	5	14.40	1.14	137.58	<0.001*
	2	5	5.40	0.55		
	3	5	7.60	0.89		
**BioRoot**	1	5	18.60	0.55	413.16	<0.001*
	2	5	6.60	1.14		
	3	5	5.60	0.55		

*P<0.05, statistically significant; P>0.05, not significant, NS

**Table 5:** Co-relation between contact angles and interfacial gap width

**Group**		**MTA**	**Biodentin**	**Bioroot**
**1**	r	-0.12	0.29	0.46
	p-value	0.84(NS)	0.63(NS)	0.44(NS)
**2**	r	0.74	-0.22	0.74
	p-value	0.15(NS)	0.72(NS)	0.15(NS)
**3**	r	0.76	0.54	-0.22
	p-value	0.13(NS)	0.35(NS)	0.72(NS)

Pearson’s correlation test *p<0.05 statistically significant, p>0.05 Non Significant, NS

**Figure 4:**
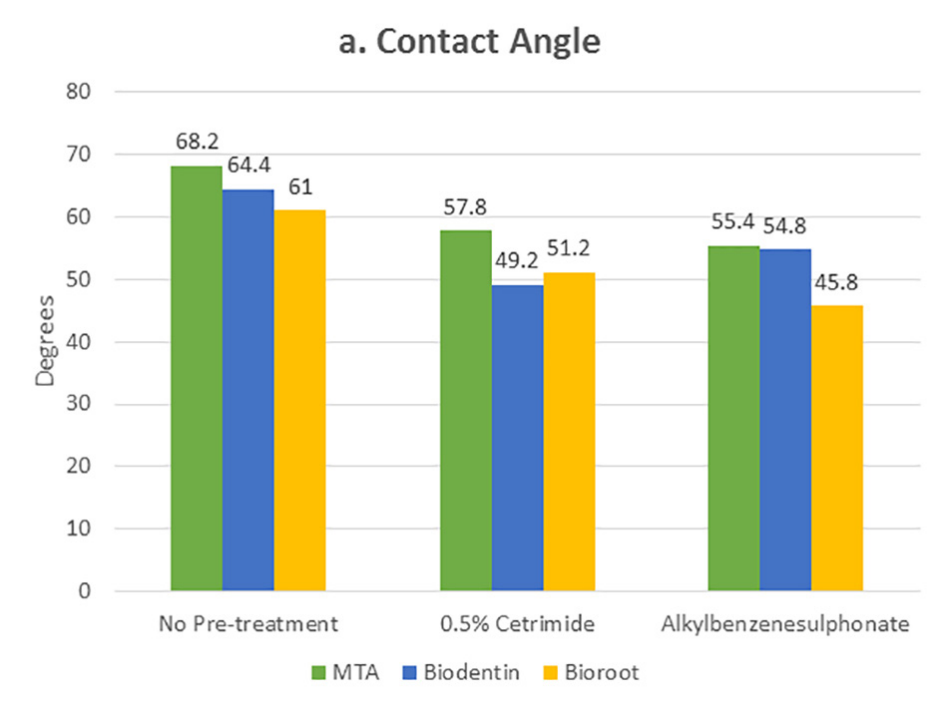
Comparison of mean contact angle values after different pre-treatments on root canal dentin surface filled with different materials.

**Figure 5:**
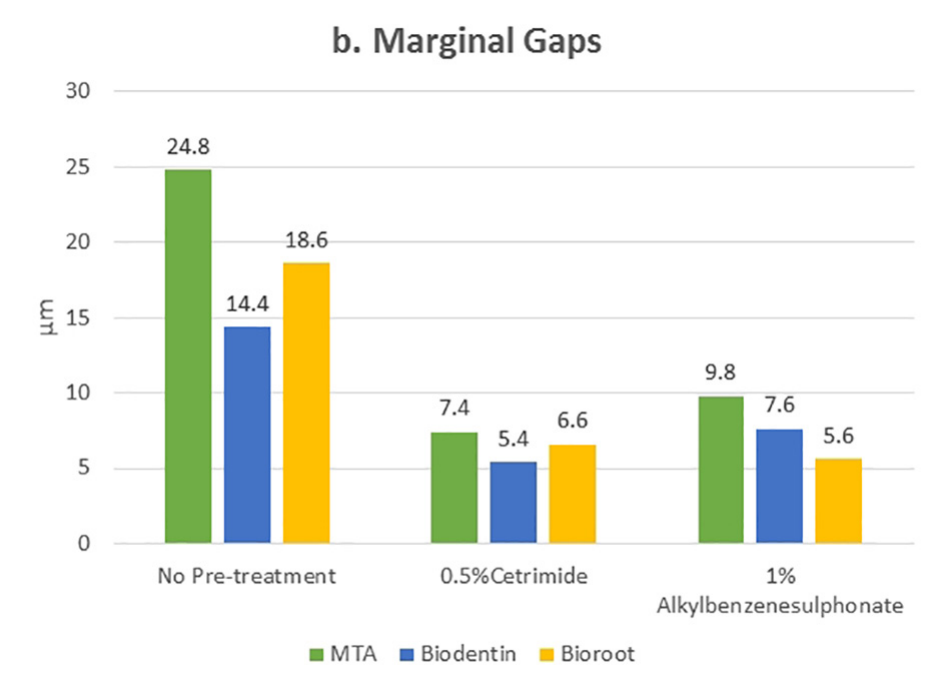
Comparison of mean values of the interfacial gap width after different pre-treatments on root canal dentin surface filled with different materials

**Figure 6:**
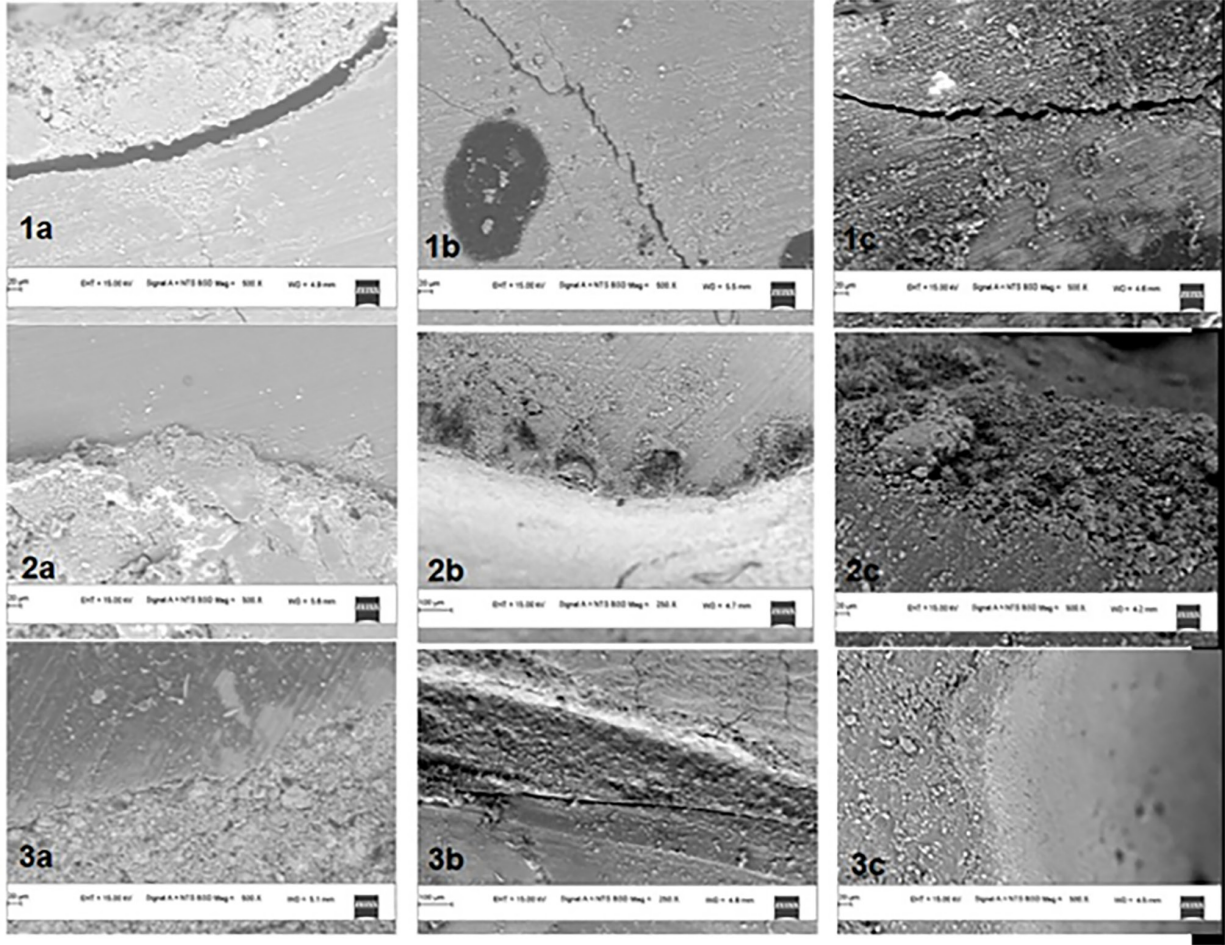
SEM images of marginal adaptation of bioceramics after dentin surface pre-treatment (1: No pre-treatment; 2: 0.5% cetrimide; 3: 1% alkylbenzene sulfonate; a: MTA; b: Biodentine; c: BioRoot).

Statistically significant values were recorded for cetrimide and alkylbenzene sulfonate compared to the no-pre-treatment group (Figures 4 and 5). For all the three materials, the interfacial gap width values were significantly lower for the surface-treated groups compared to the no-pre-treatment groups (Figure 6). No correlation was seen between the contact angle and the marginal gap.

## Discussion

Bioceramics can exhibit high bioactivity only when they interact adequately with the dentin surface, i.e., the wettability should be adequate. Contact angle evaluation is a suitable indicator of the wettability of a substance.^3-6^ The results of the present study demonstrated that cetrimide and alkylbenzene sulfonate reduced the contact angle and increased the marginal adaptation of MTA, Biodentine, and BioRoot compared to the no-pre-treatment group. In other words, samples without any pre-treatment exhibited high contact angle values, i.e., low wettability and increased interfacial gap width. In the no-pre-treatment group, MTA exhibited the poorest wettability compared to Biodentine and BioRoot, which might be attributed to two main factors: particle size and chemical composition. MTA consists of large coarse particles that prevent its adhesion to the dentinal surface. Moreover, there are slight changes in the composition, which leads to poor micro-tag formation compared to Biodentine and BioRoot, where a smoother and more delicate structure can be seen.^20,21^

Samples pretreated with 0.5% cetrimide for one minute exhibited improved wettability and marginal adaptation, especially for Biodentine. Cetrimide belongs to a group of surfactants that are surface-active agents consisting of both hydrophobic and hydrophilic groups; hence, they are called amphiphilic molecules. At the interface, they align themselves so that the hydrophobic part is in the air, and the hydrophilic part is in water, leading to a decrease in surface or interfacial tension. Chemically, cetrimide (CTAB) is a quaternary ammonium compound which is categorized under the class of cationic surfactants with a net positive charge; the ammonium bromide being hydrophilic increases the surface energy of root dentin and hence improves its wettability.^22^ It has been used as an effective antibacterial agent previously but not as a surfactant alone.^23^

Samples pretreated with 1% alkylbenzene sulfonate solution for one minute exhibited reduced contact angle values and interfacial gap width, especially for BioRoot. Alkylbenzene sulfonate belongs to a group of anionic surfactants where sulfonate is the hydrophilic part, which is responsible for reducing the surface tension. It works by increasing the surface energy and enhancing the adaptation of the material like a typical surfactant; however, there is insufficient data for its use on root dentin.^19^ Finally, no correlation was found between wettability and marginal adaptation.

A controlled volume (0.1 mL) of each material was dispensed for measuring the contact angle to prevent any volumetric change so that the value of contact angle measurement was not affected. The root dentin was dried with paper points before the placement of the material for contact angle measurement and scanning electron microscopic analysis.

The limitations of this study include the questionable cytotoxicity of alkylbenzene sulfonate since, to date, no other studies have used this surfactant. Also, if the cetrimide solution is not appropriately diluted, it decreases dentin microhardness when used at concentrations >0.5%.^24^ Hence, further studies are necessary to evaluate the clinical application of this novel approach.

## Conclusion

In conclusion, the present study showed that samples subjected to pre-treatment exhibited significantly better wettability and marginal adaptation. Specifically, samples treated with 0.5% cetrimide for one minute showed significantly better values for Biodentine, and samples treated with 1% alkylbenzene sulfonate for one minute showed significantly better values for BioRoot. Samples with no pre-treatment exhibited the poorest wettability and marginal adaptation. Wettability of the materials was not directly related to their marginal adaptation.

## Authors’ Contributions

MNwas responsible for the concept and design of the work.SQcollected,analyzed, and interpreted the data, and drafted the manuscript. DD was responsible for the experiment design and contributed to the discussion. MY revised the manuscript critically.

## Acknowledgments

The authors would like to thank Dr. Urvashi Sodvadiya and Dr. Mrinalini, who helped in the preparation of the samples. We also wish to thank Dr. Vinayak Kamat and Mr. Murari for their assistance in statistical analysis and SEM examination of this study.

## Funding

Not applicable.

## Competing Interests

The authors declare no competing interest with regards to the authorship and publication of this article.
